# Degradation of Artemisinin-Based Combination Therapies under Tropical Conditions

**DOI:** 10.4269/ajtmh.15-0665

**Published:** 2016-05-04

**Authors:** Zoe Hall, Elizabeth Louise Allan, Donelly Andrew van Schalkwyk, Albert van Wyk, Harparkash Kaur

**Affiliations:** Department of Clinical Research, Faculty of Infectious and Tropical Diseases, London School of Hygiene and Tropical Medicine, London, United Kingdom; Department of Immunology and Infection, Faculty of Infectious and Tropical Diseases, London School of Hygiene and Tropical Medicine, London, United Kingdom

## Abstract

Poor quality antimalarials, including falsified, substandard, and degraded drugs, are a serious health concern in malaria-endemic countries. Guidelines are lacking on how to distinguish between substandard and degraded drugs. “Forced degradation” in an oven was carried out on three common artemisinin-based combination therapy (ACT) brands to detect products of degradation using liquid chromatography mass spectrometry and help facilitate classification of degraded drugs. “Natural aging” of 2,880 tablets each of ACTs artemether/lumefantrine and artesunate/amodiaquine was undertaken to evaluate their long-term stability in tropical climates. Samples were aged in the presence and absence of light on-site in Ghana and in a stability chamber (London), removed at regular intervals, and analyzed to determine loss of the active pharmaceutical ingredients (APIs) over time and detect products of degradation. Loss of APIs in naturally aged tablets (both in Ghana and the pharmaceutical stability chamber) was 0–7% over 3 years (∼12 months beyond expiry) with low levels of degradation products detected. Using this developed methodology, it was found that a quarter of ACTs purchased in Enugu, Nigeria (concurrent study), that would have been classified as substandard, were in fact degraded. Presence of degradation products together with evidence of insufficient APIs can be used to classify drugs as degraded.

## Background

Malaria, caused by parasites of the genus *Plasmodium*, is a major public health burden in the tropics resulting in 584,000 deaths in 2013.^1^ Artemisinin-based combination therapies (ACTs,) are the mainstay of the treatment used in the majority of malaria-endemic countries and have greatly contributed to the reduction in malaria morbidity and mortality.^2^ Artemisinin is extracted from the plant *Artemisia annua*, generally known as sweet wormwood.^3^,^4^ Chemical derivatives of artemisinin (artesunate [AS], artemether [AM], and dihydroartemisinin [DHA]) are combined with a partner drug such as amodiaquine (AQ), lumefantrine (LUM), or piperaquine (PIP) to form currently used ACTs: AS/AQ, AM/LUM, and DHA/PIP.^5^,^6^

Efficacious malaria control and treatment necessitates the use of good quality medication that contains the required dose of active pharmaceutical ingredients (APIs). Studies predominantly in Africa and southeast Asia have suggested that a significant proportion of ACTs are of poor quality,^7^–^10^ which poses a threat to malaria patients as a result of ineffectual treatment^11^ and could facilitate the emergence and spread of drug-resistant parasites.^12^ Poor quality drugs include substandard drugs (these contain either less than or more than the acceptable dose of APIs as a result of poor manufacturing practices), falsified (also referred to as counterfeit or spurious medicines; do not contain the stated APIs and may carry false representation of their source of identity) and degraded formulations.^13^,^14^ Degraded drugs are defined as good quality formulations that are degraded by storage in the presence of heat, light, and humidity after they leave the manufacturer. Factors postulated to contribute to the degradation of a drug include ambient temperature, moisture, light, microbes, packaging materials, transportation conditions, and the nature of the active ingredients and excipients.^15^

The assessment of the prevalence of substandard medicines is hindered by the lack of information regarding the stability of medicines that may have been of acceptable quality when released by the manufacturer and have since deteriorated during transportation. Of concern is the intrinsic stability of artemisinin derivatives under conditions of high temperature and humidity, typical in tropical countries where malaria is endemic.^16^,^17^ The International Conference on Harmonization (ICH) and the World Health Organization (WHO) state that to fully assess the long-term stability of finished pharmaceutical products, testing should be conducted during and beyond the expected shelf life and storage conditions experienced in the intended market.^18^ The ICH and WHO divide the globe into climatic zones depending on the prevailing annual temperature and humidity conditions, which includes the majority of malaria-endemic countries in climatic zone IV as they experience both hot and humid conditions (mean annual temperature > 22°C and relative humidity of > 45%), for which recommended conditions for stability testing are a minimum of 12 months.^18^ Stability testing by the manufacturer, however, may not reflect the actual range of temperature and humidity in uncontrolled conditions in Africa and southeast Asia. To address this, we assess the long-term stability of ACTs Coartem^®^ (AM/LUM; Novartis, China) and Winthrop^®^ (AS/AQ; Sanofi-Aventis, Morocco) over a 3-year “natural aging” study in tropical climatic conditions.

Although substandard drugs can be defined as those that do not meet the pharmacopeia-specified limits on API content, there is no established method to objectively classify drugs as degraded. Hence, in this study we aimed to develop an analytical methodology to detect the fingerprint of the degradation product profile using liquid chromatography mass spectrometry (LC–MS). “Forced degradation” of three common ACTs was carried out, and the degradation product profile was compared with drug samples after natural aging. Our findings were then used to distinguish between substandard and degraded ACT tablets purchased in Enugu, Nigeria.^19^

## Methods

### Forced degradation studies.

Three of the most common ACT formulations, 42 tablets in seven blister packs each of Coartem (AM/LUM), Winthrop (AS/AQ), and Waipa (DHA/PIP) were subjected to forced degradation^15^,^20^,^21^ in an oven set at 60°C for up to 21 days (Table 1). Half the blister packs were placed in the oven intact, whereas the other half were perforated before placement in the oven; degradation rates were compared for the two groups. Six tablets in blisters (three perforated, three intact) were removed twice per week for 3 weeks.

### Natural aging studies.

**Figure 1.**
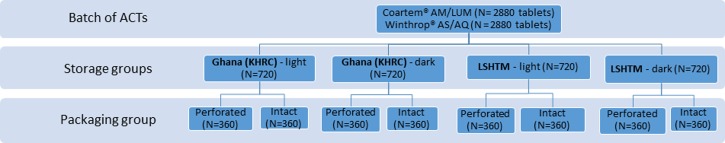
Study design for “natural aging” of ACTs. Herein, 2,880 tablets each of Coartem^®^ AM/LUM and Winthrop^®^ AS/AQ were stored for 36 months in two sites, Ghana (KHRC) and a stability chamber in London (LSHTM), in the presence and absence of light and with intact or perforated blister packs. Temperature and humidity settings in the stability chamber were adjusted on a monthly basis to the average ambient conditions in Ghana (33°C ± 5°C, relative humidity = 55% ± 20%). ACTs = artemisinin-based combination therapies; AM/LUM = artemether/lumefantrine; AS/AQ = artesunate/amodiaquine; KHRC = Kintampo Health Research Center.

The long-term stability of Coartem (AM/LUM) and Winthrop (AS/AQ) tablets was investigated in a natural aging study over 3 years by storing the tablets as intact and perforated blister packs in the presence and absence of light at tropical temperature and humidity (Table 1 and Figure 1

). Tablets were stored in their original packaging (blister pack and box) inside a drawer (dark) or on a shelf (light) at Kintampo Health Research Center, Ghana, and in the pharmaceutical stability chamber (PSC022.AHX; Weiss Gallenkamp Ltd., United Kingdom) at the London School of Hygiene and Tropical Medicine (LSHTM; Supplemental Figure 1). The temperature and humidity conditions on the stability chamber were reset on a monthly basis to correspond to field conditions recorded in Kintampo, Ghana. Over the 3 years, median temperature in Ghana was 33°C ± 5°C with a relative humidity of 55% ± 20% (typical conditions). Stability of the ACTs was determined by measuring and comparing the loss of APIs, at predetermined intervals over a 3-year period, in line with the guidelines issued by the ICH for long-term stability testing.^22^

A total of 2,880 tablets of each formulation were selected to be aged at the two sites (Figure 1), each divided into four storage groups (light perforated, light intact, dark perforated, and dark intact). At defined time points (0, 3, 6, 9, 12, 18, 24, 30, and 36 months), a total of 144 tablets of each formulation were removed from each site and stored in their blister packs at 4°C before analysis at the end of the study. All aged tablets were analyzed together at the end of the study to minimize variability in the data from changes in chromatographic conditions, and tablets at aging time 0 were used as reference. Thus, for each formulation, there were 36 replicates per storage treatment and time point, allowing a comparison between group means with a power of 80%, α = 0.05 (assuming a difference in group means of at least 3%, σ = 5%). The null hypothesis was that the API content in tablets after 36 months was not significantly different from the amount present in tablets at time 0 and that there were no significant differences between the storage groups or the condition of the packaging, that is, blister intact or perforated. Each drug was analyzed separately, and the average API content at the start of the study was compared with that after 36 months, under each of the different storage conditions, by one-way analysis of variance (ANOVA, α = 0.05). Further tests, where warranted, were performed using Tukey's honestly significant difference (HSD) post hoc tests.

### Chromatographic analyses.

The amounts of the stated APIs in the ACT tablets were quantified using high-performance liquid chromatography photo diode array (HPLC–PDA). In brief, whole tablets were pulverized and dissolved in methanol to give a final stated API (AM, AS, and DHA) concentration of 10 mg/mL and analyzed following our published method.^19^ Quantitative analyses were carried out using Dionex Ultimate 3000 HPLC–PDA system (Thermo Fisher, Hemel Hempstead, United Kingdom), and separation was achieved using a GENESIS^®^ AQ 4-μm column (150 × 4.6 mm; Grace Materials Technologies, Carnforth, United Kingdom).^23^ Quality-assured APIs were used for the calibration plots; AS and AQ were a gift from Sanofi-Aventis Group (Gentilly Cedex, France), and AM and LUM were a gift from Novartis Pharma AG (Basel, Switzerland).

The LC–MS method was developed to obtain the chromatographic “fingerprint” or “profile” of the drug(s) and their specific degradation products using Dionex Ultimate 3000 LC system coupled to Thermo Finnigan LCQ Advantage (Thermofisher). Samples were separated on a Dionex Acclaim 120 3-μm C18 column (4.6 × 150 mm) with isocratic elution at a flow rate of 1 mL/minute. Mobile phase for AM/LUM consisted of 90/10 (v/v) methanol/ammonium formate buffer (10 mM, pH 2.7), AS/AQ required 65/35 (v/v) acetonitrile/ammonium formate buffer, and DHA/PIP samples were eluted with 75/25 (v/v) methanol/ammonium formate buffer. Ions were generated by electrospray ionization and the mass spectrometer operated in positive ion mode. Spray and tube lens offset voltages were set at 5 kV and 20 V, respectively. The capillary voltage was set to 16 V (AM) or 3 V (AS and DHA). The capillary temperature was 200°C, and sheath and auxiliary gas (nitrogen) flow rates were 70 and 10 arbitrary units (AM) or 50 and 10 arbitrary units (AS and DHA).

Mass spectra were measured and compared for the artemisinin derivatives (Supplemental Figure 2) and their degradation products (Supplemental Figure 3), arising after forced degradation. Several compounds that were hypothesized to result from the degradation of ACTs were synthesized a priori including 9,10-anhydroartemisinin, 2-deoxyartemisinin, β-AS, peroxyhemiacetal, deoxy DHA, and α-AM.^22^,^23^ These products were analyzed by LC–MS and considered as a match if both retention time and mass spectra were the same as those of the unknown degradation products generated by the process of forced degradation. Fractions identified to contain the degradation products were collected using semi-preparative separation, freeze dried, and used to identify degradation products in the “naturally aged” ACTs as well as to test for antiplasmodial activity.

### Antiplasmodial activity of degradation products.

The antiplasmodial activity of the degradation products (D1–D4) resulting from the artemisinin derivatives were investigated in vitro and compared with the activity of artemisinin standards (AS, AM) using two recent African *Plasmodium falciparum* isolates, according to standard methods described by van Schalkwyk and others^24^ (Supplemental Information).

### Differentiation of substandard and degraded ACTs from Enugu.

Samples of ACT tablets that had been recently purchased for a drug quality survey in Enugu, and classified as substandard after laboratory analyses for drug content^19^ were reanalyzed using the LC–MS method developed here to investigate the presence of degradation products. Degradation product profiles were used to differentiate between substandard and degraded drugs. ACT tablets were kept at room temperature and analyzed for degradation products within 4 weeks of analysis for drug content by HPLC–PDA.

## Results

### Forced degradation studies.

Blister packs of AS/AQ, AM/LUM, and DHA/PIP tablets were subjected to degradation in an oven at 60°C for up to 21 days and analyzed by LC–MS. The resulting chromatograms were compared with those obtained from the analysis of quality-assured tablets that had not been subjected to degradation.

#### Degradation of AS/AQ tablets.

**Figure 2.**
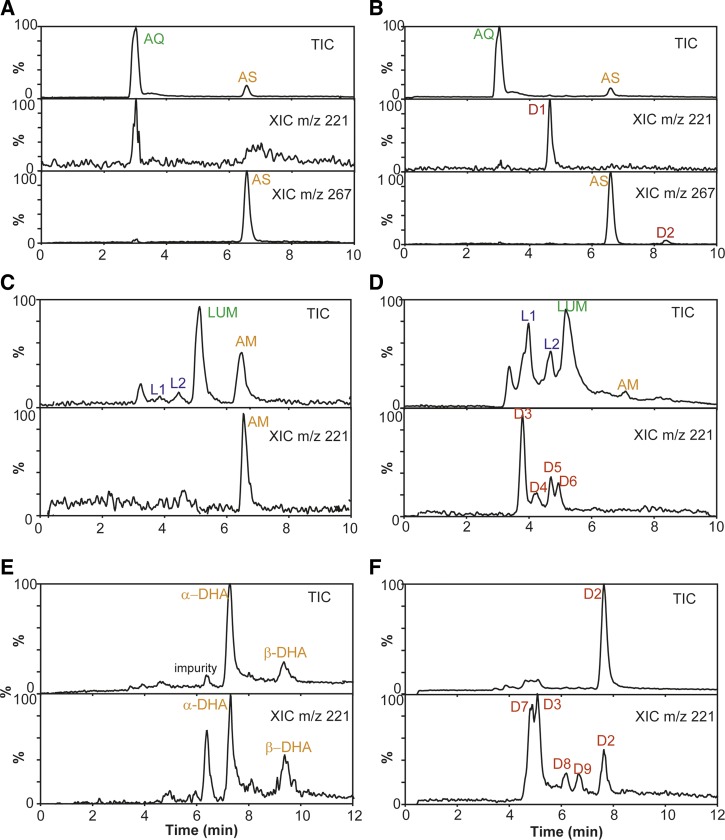
Artemisinin-based combination therapies before and after “forced degradation” at 60°C. AS/AQ tablets analyzed by liquid chromatography mass spectrometry (LC–MS) before (**A**) and after (**B**) degradation. Degradation products D1 and D2 were observed by extracted ion chromatograms (XIC) at *m/z* 221 and *m/z* 267. AM/LUM tablets analyzed by LC–MS before (**C**) and after (**D**) degradation. Degradation products D3–D6 were observed for AM, while LUM degradation products L1 and L2 were identified as desbutylketo derivatives of LUM (L1) and LUM-N-oxide (L2). DHA/PIP tablets analyzed by LC–MS before (**E**) and after (**F**) degradation. Degradation products D2, D3, and D7–D9 were observed by extracting chromatograms at *m/z* 221. AM/LUM = artemether/lumefantrine; AS/AQ = artesunate/amodiaquine; DHA/PIP = dihydroartemisinin/piperaquine.

**Figure 3.**
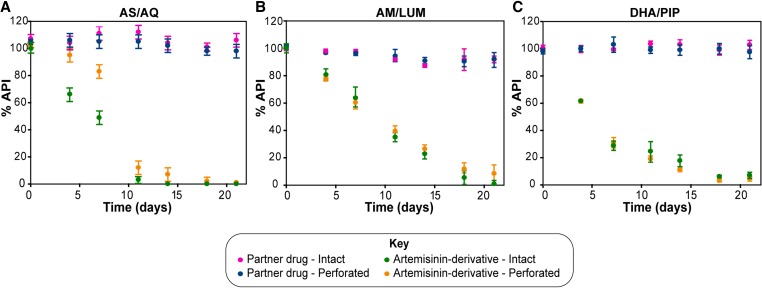
Degradation over time for “forced degradation” of artemisinin-based combination therapies. Forced degradation of (**A**) AS/AQ, (**B**) AM/LUM, and (**C**) DHA/PIP tablets. Tablets were sampled twice a week, and the %API for the partner drugs (AQ, LUM, and PIP) and artemisinin derivatives (AS, AM, DHA) was quantified using high-performance liquid chromatography photo diode array. Error bars show standard deviation (*N* = 3). AM/LUM = artemether/lumefantrine; %API = active pharmaceutical ingredient; AS/AQ = artesunate/amodiaquine; DHA/PIP = dihydroartemisinin/piperaquine.

Peaks corresponding to the AS and AQ components (LC retention time 6.5 and 3.0 minutes, respectively) were identified in tablets before and after degradation (Table 2, Figure 2A and B

). In the degraded tablets, additional peaks from two degradation products of AS—D1 and D2 (retention times 4.7 and 8.3 minutes, respectively)—were detected by extracting chromatograms at *m/z* 221 and *m/z* 267, common signature ions for fragments of fragile artemisinin derivatives (Table 2 and Figure 2B).^26^,^27^ On comparison with standards of possible degradation products, the only compound identified was 2-deoxyartemisinin (D2; Supplemental Figure 4). The API content in the co-formulated AS/AQ tablets (bilayer tablets) was monitored for 21 days. Although AQ content was stable over this period, AS content decreased over time. Packaging (intact or perforated) did not affect the amount of AQ, whereas the amount of AS decreased more rapidly when the packaging was intact compared with when the tablets were exposed to the atmosphere in perforated packaging (Figure 3A

).

#### Degradation of AM/LUM tablets.

Before degradation, the chromatograms (Table 2 and Figure 2C) were characterized by peaks corresponding to LUM and AM (retention times 5.2 and 6.6 minutes, respectively). Additional peaks were noted in the degraded tablets at retention times 3.2, 3.8, and 4.5 minutes (Table 2 and Figure 2C and D). The latter two of these peaks (L1 and L2) were identified from the literature as LUM impurities.^28^–^30^ Only small amounts of AM remained in the degraded tablets (Figure 2D); by extracting chromatograms at *m/z* 221, four additional peaks were observed, which were assigned to AM degradation products D3–D6 (Figure 2D). The primary AM degradation product observed was D3 with a retention time of 3.9 minutes (Table 2). On comparison with standards, the only compounds identified were desbutylketo derivatives of LUM (L1) and LUM-N-oxide (L2). Monitoring the API content over time, showed LUM to be stable for 21 days, whereas AM content decreased. Packaging (intact or perforated) did not affect the AM or LUM content under forced degradation (Figure 3B).

#### Degradation of DHA/PIP tablets.

DHA is considered to be the least stable artemisinin derivative formulated in ACTs.^16^,^31^ Before degradation (Figure 2E and Table 2), chromatographic peaks were observed, which corresponded to the two epimers of DHA (α- and β-DHA, retention times 7.2 and 9.3 minutes, respectively). In addition, a further peak, accepted as an impurity or degradation product of DHA, was observed (retention time 6.3 minutes; Figure 2E). After forced degradation, the DHA peaks diminished indicating the complete loss of this API. A large peak was observed with an identical retention time to α-DHA (Figure 2F); however, its mass spectrum matched that of D2 and not DHA (Supplemental Figures 2 and 3). A further four peaks (Figure 2F) were detected; three of these were assigned as DHA degradation products D7–D9 (Table 2). The fourth peak (retention time 5.0 minutes) had a mass spectrum identical to that of D3 (Supplemental Figure 3), also a product of AM degradation. By comparison with standards of possible degradation products, the only compound identified was 2-deoxyartemisinin (D2). API content over time was measured; as with previous ACTs, the partner drug PIP did not change with time, whereas DHA content decreased substantially. Packaging (intact or perforated) did not affect the amount of DHA or PIP in these co-formulated DHA/PIP tablets (Figure 3C).

### Antiplasmodial activity.

The antiplasmodial potency of the major degradation products D1–D4 were tested against *P. falciparum* parasites in vitro and compared with that of AS and AM.^24^ The assays (Supplemental Table 1) revealed that AS and AM exhibit potent antimalarial properties as expected, whereas the degradation products D1–D4 and 2-deoxyartemisinin have an almost 1,000-fold or greater reduction in antimalarial activity.

### Natural aging: Stability of ACTs in tropical climatic conditions.

This study undertook to conduct natural aging of two commonly used ACTs from WHO prequalified manufacturers,^25^ AS/AQ and AM/LUM, over 3 years at tropical temperature and humidity (Table 1). Tablets of each formulation were naturally aged in two sites and under four storage conditions (Figure 1), and their API content was monitored with time.

**Figure 4.**
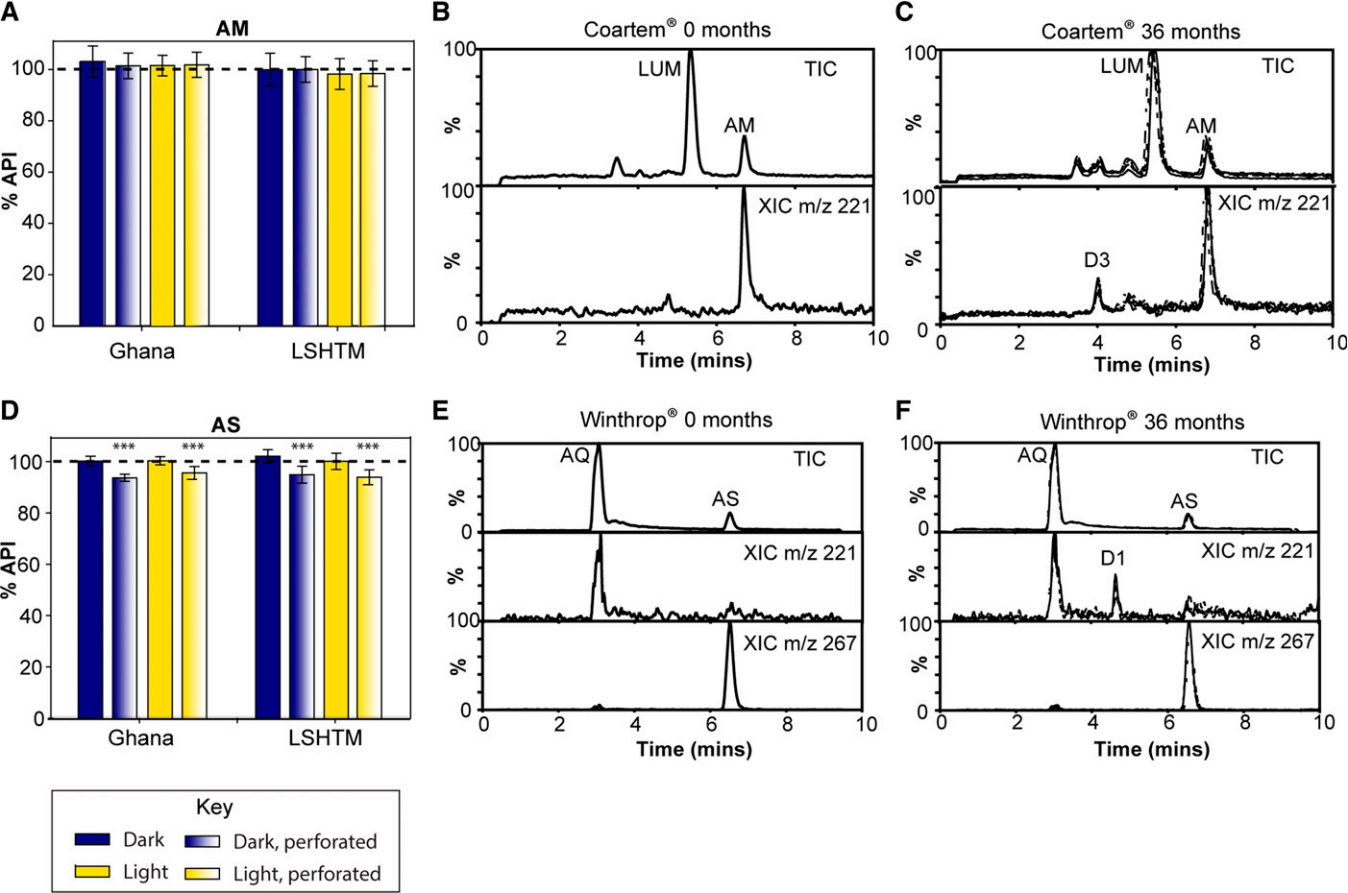
Long-term stability of artemisinin-based combination therapies in tropical climates. AM content in AM/LUM tablets (**A**) and AS content in AS/AQ tablets (**D**) was measured at 36 months after storing at high temperature and humidity in Ghana or a pharmaceutical stability chamber (LSHTM). Error bars represent standard deviation (*N* = 36). AS/AQ tablets in perforated blister packs showed significantly lower levels of AS than those in which the blister packaging was intact (*** *P* < 0.001). Tablets were analyzed by liquid chromatography mass spectrometry and representative chromatograms are shown at the start (**B**, **E**) and end (**C**, **F**) of the study. Extracted chromatograms (*m/z* 221) show the presence of degradation product D3 in aged AM/LUM and D1 in aged AS/AQ tablets. AM/LUM = artemether/lumefantrine; AS/AQ = artesunate/amodiaquine; DHA/PIP = dihydroartemisinin/piperaquine; LSHTM = London School of Hygiene and Tropical Medicine.

The amount of partner drugs AQ and LUM in the co-formulated AS/AQ and AM/LUM (Supplemental Figure 5) was found to be stable for at least 3 years under the storage conditions used (*F*_8,315_ < 1.96, *P* > 0.05). Similarly, the ANOVA of AM concentrations revealed no significant differences between the start and end of the study (*F*_8,315_ = 1.73, *P* > 0.05) for any of the storage groups. This suggests that AM content did not vary over the duration of the study or between different storage groups (Figure 4A

and Supplemental Figure 6).

In contrast, a significant difference in AS content of the AS/AQ tablets was observed between the different groups at 36 months and the start of the study (*F*_8,315_ = 30.5, *P* < 0.001). Post hoc comparisons using the Tukey's HSD test revealed that significantly lower levels of AS (decrease of 5–7%) were measured at the end of the study for tablets that had been stored in perforated blister packs (*P* < 0.001; Figure 4D and Supplemental Figure 6), whereas other storage groups did not differ significantly in their AS content from the initial values. To further investigate the effect of storage conditions on AS content after 36 months, a three-factor ANOVA (site, lighting, and packaging) showed no significant main effect for the site (LSHTM/Ghana) factor (*F*_1,280_ < 1, nonsignificant [ns]), no significant main effect for the lighting factor (*F*_1,280_ < 1, ns); however, the main effect for the packaging factor (perforated/intact) was highly significant (*F*_1,280_ = 62, *P* < 0.001). There were no significant interaction effects.

Although the amount of AM did not change significantly from its initial amount, the presence of a degradation product was detected by MS in tablets after 36 months, albeit at low levels, which was not present initially (Figure 4B and C). Similarly, a degradation product was observed in naturally aged AS/AQ tablets after 36 months (Figure 4E and F). These degradation products had matching retention times and mass spectra to those of D3 and D1, respectively, which were also observed in the forced degradation study of AM/LUM and AS/AQ tablets at 60°C.

### Differentiation between substandard and degraded ACT formulations purchased in Enugu.

Samples of ACT tablets purchased in Enugu, and previously found to be substandard with APIs of < 85% were reanalyzed (*N* = 44) using LC–MS to test for degradation products.^19^ Suspensions were excluded from this reanalysis. The tablets of ACTs included various AM/LUM brands (Amatem Forte^®^, Amatem Tab^®^, Arcofan, Artrin^®^, Artemetrin^®^, Fynale, Ogamal, and Ogamal QS), one AS monotherapy brand (Maltarka), and one DHA/PIP brand (Droa-Quine^®^). Within the brands identified to contain degradation products, 38 packages of 152 (25.0%) were found to contain degraded tablets, six (3.9%) contained substandard tablets, and 108 (71.1%) were of good quality (Table 3). The only brands for which all the packages examined (although in some cases we only had one package) and that contained tablets with degradation products were Arcofan, Fynale, Ogamal, and Droa-Quine^®^, whereas in other brands, a proportion of the packages revealed evidence of products of degradation (Table 3).

**Figure 5.**
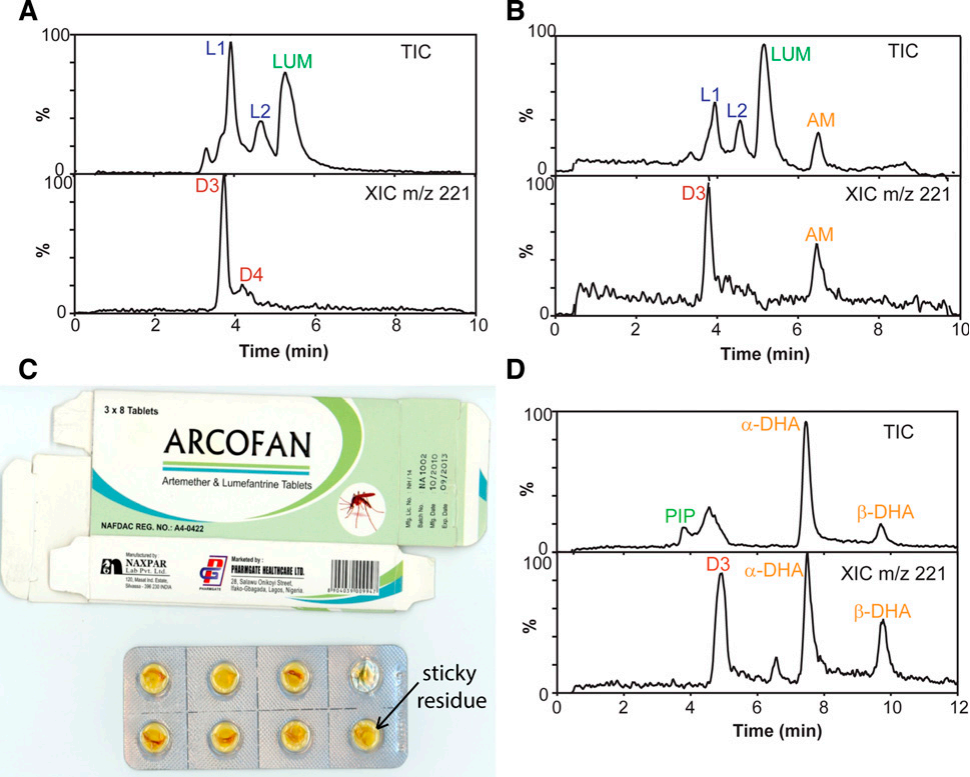
Comparing degraded tablets (field samples) with artificially degraded tablets. Liquid chromatography mass spectrometry (LC–MS) analysis of AM/LUM Arcofan (**A**) and Amatem Tab^®^ (**B**) tablets revealed similar degradation products to artificially degraded AM/LUM tablets (“forced degradation”). Extracted ion chromatograms for *m/z* 221 (signature fragment ion) are shown below the total ion chromatogram. Examination of the Arcofan packaging revealed a sticky residue on the inside of the blister packs (**C**). LC–MS analysis of Droa-Quine^®^ DHA/PIP tablet revealed the presence of degradation product D3 (**D**). AM/LUM = artemether/lumefantrine; DHA/PIP = dihydroartemisinin/piperaquine.

The major degradation products that were isolated from the AM/LUM tablets after forced degradation were D3 and D4. These degradation products were also observed in the majority of substandard AM/LUM field tablets (aka field samples) from Enugu (two examples are shown in Figure 5A and B

, and further examples are provided in the Supplemental Appendix). Two degradation products of LUM were also detected, desbutylketo derivatives of LUM (L1) and LUM-N-oxide (L2). In addition, package analysis revealed that some of the tablets were discolored and/or there was presence of a sticky residue (Figure 5C). Similarly, Droa-Quine^®^ DHA/PIP tablets purchased in the field and determined to contain a low %DHA by HPLC–PDA were reanalyzed by LC–MS (Figure 5D) and found to contain DHA degradation product D3 (matching retention time and mass spectrum).

## Discussion

This study demonstrates that the use of fingerprint chromatographic profiles obtained by subjecting tablets to forced degradation can be used to facilitate the classification of drugs as degraded. Furthermore, the forced degradation study revealed that the artemisinin derivatives AS, AM, and DHA degrade extensively when stored at 60°C for up to 21 days, while partner drugs AQ, LUM, and PIP were stable under these conditions. The integrity of the packaging, that is, whether the blister pack was perforated or intact, did not affect the forced degradation of AM and DHA; however, AS degraded more rapidly in the intact blister packs. The instability of AS (dihydroartemisinin-10α-hemisuccinate) in the presence of AQ (present here as the dihydrochloride salt) is well documented, particularly at high heat and humidity.^32^,^33^ In this forced degradation study, moisture was noticed to be retained in the intact blisters, whereas tablets stored in perforated blister packs were found to be dry and brittle. One of the main degradation products of AS was identified as 2-deoxyartemisinin, but the identity of the other products is beyond the scope of this study. Degradation products were isolated and found to have negligible antiplasmodial potency against two recent African isolates of *P. falciparum*, indicating that severely degraded ACTs will be ineffectual for malaria treatment.

Tablets of Coartem AM/LUM and Winthrop AS/AQ (common brands of ACTs from WHO prequalified manufacturers) were found to be stable when naturally aged under tropical conditions. Acceptable levels of all APIs (90–110% as per International Pharmacopeia tolerance limits) were measured over 3 years, despite drugs having reached their expiry dates 18 to within 24 months from the start of the study. This suggests that the shelf life of AM/LUM and AS/AQ could be reevaluated in an effort to improve the cost-effectiveness of the treatment in the poorest countries.^34^ Although no significant degradation was observed for AM, LUM, and AQ over the study period, low levels of degradation (< 7%) for AS was measured for tablets stored in perforated blister packs. This suggests that the degradation for AS in co-formulated AS/AQ tablets is higher when the tablets are exposed directly (perforated blisters) to conditions of high heat and humidity. Low levels of degradation products could be detected in tablets from both ACT formulations after 18 months, demonstrating that the LC–MS method reported here is a sensitive and robust approach to establish the presence of degradation products in ACTs, even before substantial loss of the API has occurred. Reanalyses of the tablets from Enugu, initially classified as substandard (when they contained < 85% APIs), revealed 25.0% to be degraded (Table 3), suggesting that degradation of ACTs may account for a high proportion of drugs previously classified as substandard or indeed those suspected to be falsified since several degraded AM/LUM field samples had no detectable amounts of AM (although levels of LUM were within the acceptable range).

The natural aging study indicates that quality-assured drugs from WHO prequalified manufacturers are stable in tropical climates for periods up to and well beyond their expiry dates. However, some brands of tablets purchased in Enugu reclassified to be degraded were from non-WHO prequalified manufacturers. This observation warrants further investigation to determine if all brands of ACTs are stable in tropical climates.

## Conclusion

Poor quality medications are of great concern and may include falsified, substandard, and degraded drugs. In this study, three common ACTs were subjected to forced degradation and an LC–MS method was developed to aid the differentiation of substandard and degraded drugs. Application of this method to a large number of tablets purchased in Enugu revealed that degradation was one of the major hitherto not recognized causes of drugs failing chemical content analysis. These degraded ACTs were from manufacturers without WHO prequalification status. However, our findings in a large-scale natural aging study showed that quality-assured ACTs from WHO prequalified manufacturers were stable in tropical conditions of high temperature and humidity for over 3 years, despite drugs reaching their expiry dates before the end of the study. This stability was also observed within the ACTs purchased in Enugu, which were stated to be from WHO prequalified manufacturers. Although these findings cannot be generalized, they do warrant further investigation of drug stability in a variety of settings in malaria-endemic countries. Furthermore, manufacturers are to be encouraged to attain WHO prequalification status, and follow-up studies are warranted to uncover factors that may have an impact on drug stability. Degraded drugs should be differentiated from substandard drugs as those that do not comply with the pharmacopeia-specified acceptance criteria of containing less than the acceptable API content and in which products of degradation are detected.

## Supplementary Material

Supplemental Datas.

## Figures and Tables

**Table 1:** Study details for “forced degradation” and “natural aging” of artemisinin-based combination therapies

Location of study	Formulation and manufacturer	Manufacture information
Forced degradation		
60°C oven (LSHTM) Study date October 2013 to December 2013	Winthrop^®^ (100 mg AS/270 mg AQ) Sanofi-Aventis Group, Maphar Laboratories, Morocco	Lot 5491 Expiry: May 2015
	Coartem^®^ (20 mg AM/120 mg LUM) Novartis, China	Lot X1607 Expiry: September 2014
	Waipa (30 mg DHA/225 mg PIP) Kunimed Pharmachem Ltd., Nigeria	Lot SFH08 Expiry: December 2013
Natural aging		
Ghana (KHRC) Study date January 2011 to January 2014	Winthrop^®^ (100 mg AS/270 mg AQ) Sanofi-Aventis Group, Maphar Laboratories, Morocco	Lot 5186 Expiry: June 2012
	Coartem^®^ (20 mg AM/120 mg LUM) Novartis, China	Lot X1448 Expiry: December 2012
Stability chamber (LSHTM)	Winthrop^®^ (100 mg AS/270 mg AQ) Sanofi-Aventis Group, Maphar Laboratories, Morocco	Lot 5186 Expiry: June 2012
	Coartem^®^ (20 mg AM/120 mg LUM) Novartis, China	Lot X1448 Expiry: December 2012

AM = artemether; AS = artesunate; AQ = amodiaquine; DHA = dihydroartemisinin; KHRC = Kintampo Health Research Center; LSHTM = London School of Hygiene and Tropical Medicine; LUM = lumefantrine; PIP = piperaquine.

**Table 2:** Summary of degradation products identified by liquid chromatography mass spectrometry after “forced degradation” of artemisinin-based combination therapies in an oven at 60°C for up to 21 days

Formulation	API (retention time)	Degradant (retention time)	Figure (chromatogram/spectrum)
AS/AQ Winthrop^®^	AS (6.5 minutes); AQ (3.0 minutes)	D1 (4.7 minutes); D2 (8.3 minutes)	Figure 2A and B/Supplemental Figures 2 and 3
AM/LUM Coartem^®^	AM (6.6 minutes); LUM (5.2 minutes)	L1 (3.8 minutes); L2 (4.5 minutes); D3 (3.9 minutes); D4-D6 (4.0–5.2 minutes)	Figure 2C and D/Supplemental Figures 2 and 3
DHA/PIP Waipa	α-DHA (7.2 minutes); β-DHA (9.3 minutes)	D2 (7.9 minutes); D3/D7 (4.5-5.5 minutes); D8 (6.2 minutes); D9 (6.6 minutes)	Figure 2E and F/Supplemental Figures 2 and 3

AM = artemether; API = active pharmaceutical ingredient; AS = artesunate; AQ = amodiaquine; DHA = dihydroartemisinin; LUM = lumefantrine; PIP = piperaquine.

**Table 3:** Proportion of acceptable quality, substandard, and degraded tablets in relation to brands of artemisinin-based combination therapies purchased in Enugu, Nigeria

Brand	Stated manufacturer	Stated APIs	Total no. of samples	Acceptable quality (%)	Substandard (%)	Degraded (%)
Amatem Forte^®^	Micro Labs Limited, India	AM/LUM	43	97.7	0	2.3
Amatem Tab^®^ 20/120	Micro Labs Limited, India	AM/LUM	31†	71.0	12.9	16.1
Arcofan 20/120	Naxpar Laboratory Pvt. Ltd., India	AM/LUM	15†	0	0	100.0
Artemetrin^®^ 80/480	A.C. Drugs Ltd., Nigeria	AM/LUM	5†	20.0	0	80.0
Artrin^®^	Medreich Limited, India	AM/LUM	15	73.3	0	26.7
Fynale	Naxpar Laboratory Pvt. Ltd., India	AM/LUM	1	0	0	100.0
Ogamal	Vapi Care Pharma Pvt. Ltd., India	AM/LUM	1†	0	0	100.0
Ogamal QS	Vapi Care Pharma Pvt. Ltd., India	AM/LUM	35	91.4	2.9	5.7
Maltarka	Vapi Care Pharma Pvt. Ltd., India	AS/S/P*	5	0	33.3	66.7
Droa-Quine^®^	Hubei Meibao Pharmaceutical Co. Ltd., China	DHA/PIP	1	0	0	100.0
Total			152 (100%)	71.1	3.9	25.0

ACTs = artemisinin-based combination therapies; AM = artemether; API = active pharmaceutical ingredient; AS = artesunate; DHA = dihydroartemisinin; LUM = lumefantrine; P = pyrimethamine; PIP = piperaquine; S = sulfadoxine.

All manufacturers listed in Table 3 were non–World Health Organization prequalified.^25^

* Not co-formulated, that is, AS in one tablet with S/P in a second tablet.

† When purchased, all tablets except one package of Ogamal had not reached their expiry date; however, three other packages of tablets (Amatem Forte^®^, Arcofan 20/120, and Artemetrin^®^ 80/480) had exceeded their expiry date at the time of laboratory analysis. All the other tablets remained within date.
