# Supplementary material for: Degradation of Artemisinin-Based Combination Therapies under Tropical Conditions
Source: Am J Trop Med Hyg. 2016 May 4;94(5):993–1001. doi: 10.4269/ajtmh.15-0665 (PMC4856632; doi:10.4269/ajtmh.15-0665)
Supplement: Supplementary file 1 [file SD2.pdf]

SUPPLEMENTAL INFORMATION

ANTIPLASMODIAL ACTIVITY ASSAY

IC_{50} estimates were obtained for the artemisinin derivatives and their degradation products for two established parasite lines, HL1210 and HL1204. In addition, IC_{50} values were estimated for artesunate (AS), artemether (AM) and two of the synthesized standards—9,10-anhydroartemisinin (epoxy bridge intact) and 2-deoxyartemisinin (epoxy bridge broken; corresponds to D2). Both parasite lines are sensitive to AS and resistant to pyrimethamine. However, HL1204 is sensitive to chloroquine, whereas HL1210 is chloroquine resistant. Therefore, AS, chloroquine, and pyrimethamine were also tested as internal controls. Each assay was performed in duplicate using 3-fold dilutions of the test compound to cover a large concentration range. Primary stock solutions for all compounds with the exception of chloroquine were made by dissolution in dimethyl sulfoxide (DMSO; Sigma Aldrich, St. Louis, MO). Subsequent dilutions were carried out in RPMI 1640 growth media (Sigma Aldrich). The DMSO present in the test concentration range was of low to negligible toxicity to the parasites. Chloroquine stock solution was prepared using deionized water (Millipore, Watford, United Kingdom).

Identification of degradation products using "forced degradation" studies. Mass spectra were examined for the artemisinin derivatives (Supplemental Figure 2) and their degradation products (Supplemental Figure 3), which were consistently observed under conditions of stress testing. Fragile artemisinin-derivative molecules undergo fragmentation in the mass spectrometer. Consequently, mass spectra are characterized by complex fragmentation patterns rather than a peak(s) corresponding to the molecular ion (plus adducts). As such, the resulting mass spectra can be informative for identification, particularly when compared with known standards.

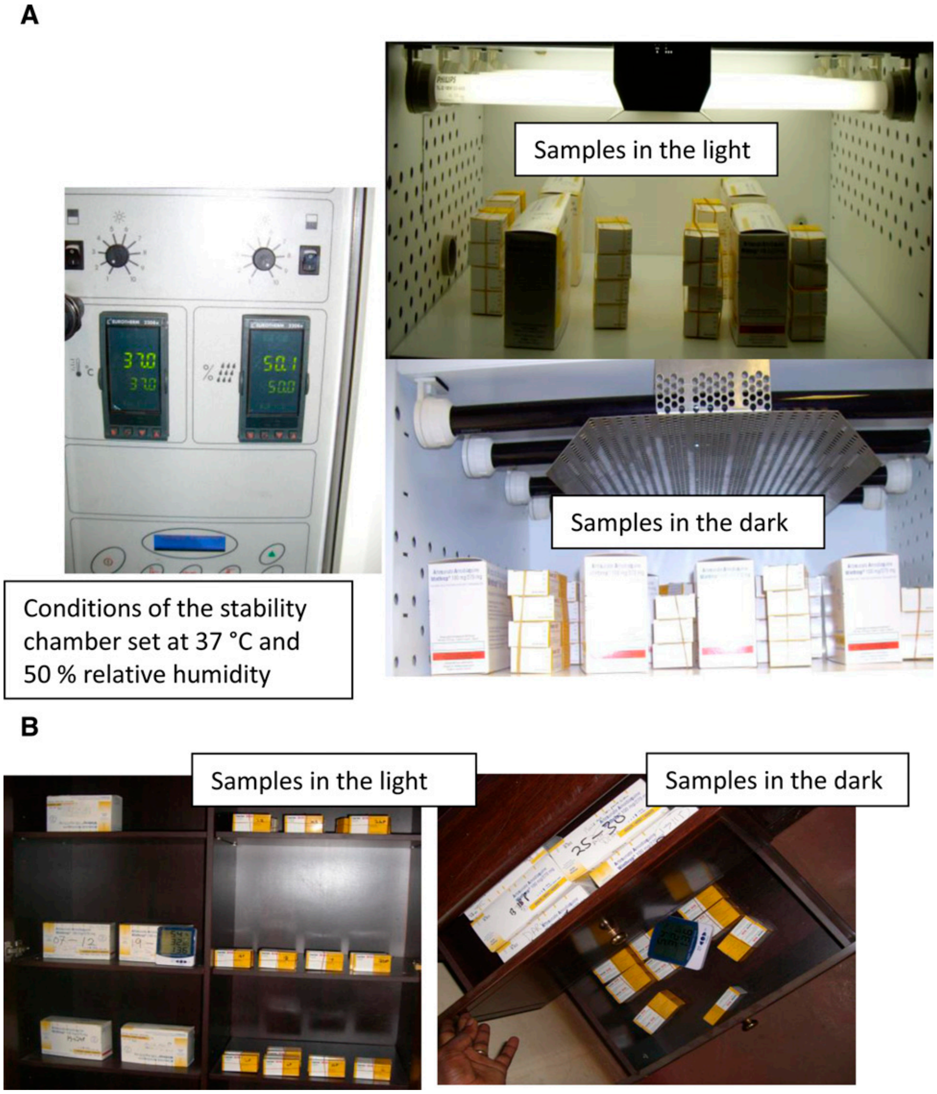

Supplemental Figure 1. Representative samples placed in the stability chamber at London School of Hygiene and Tropical Medicine (**A**) and under typical storage conditions at the Kintampo Research Health Center, Kintampo, Ghana (**B**).

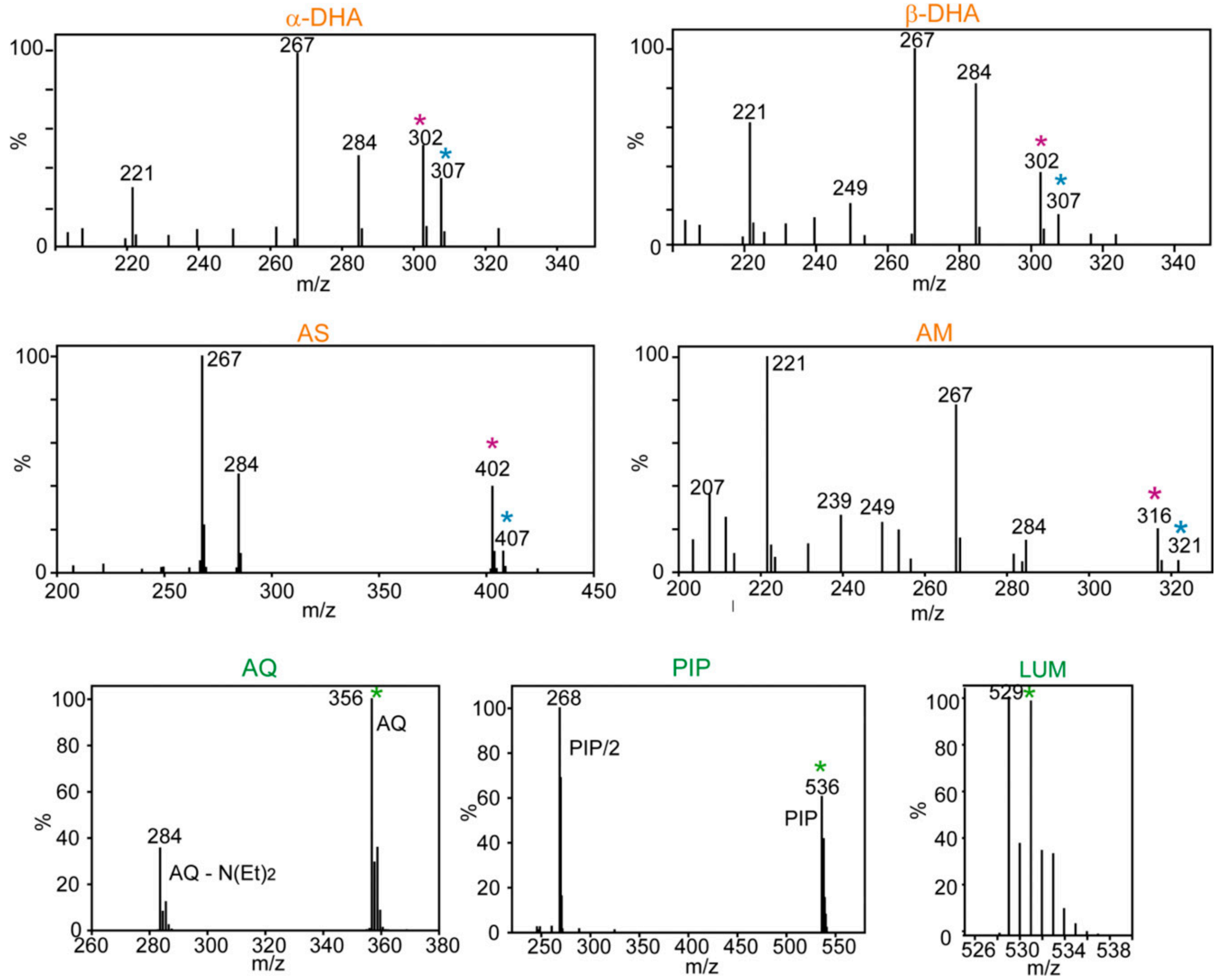

Supplemental Figure 2. Mass spectra for artemisinin derivatives (orange text) and their partner drugs (green text). Starred peaks indicate the molecular ion + H^+ (green), + NH_4^+ (pink), and + Na^+ (blue).

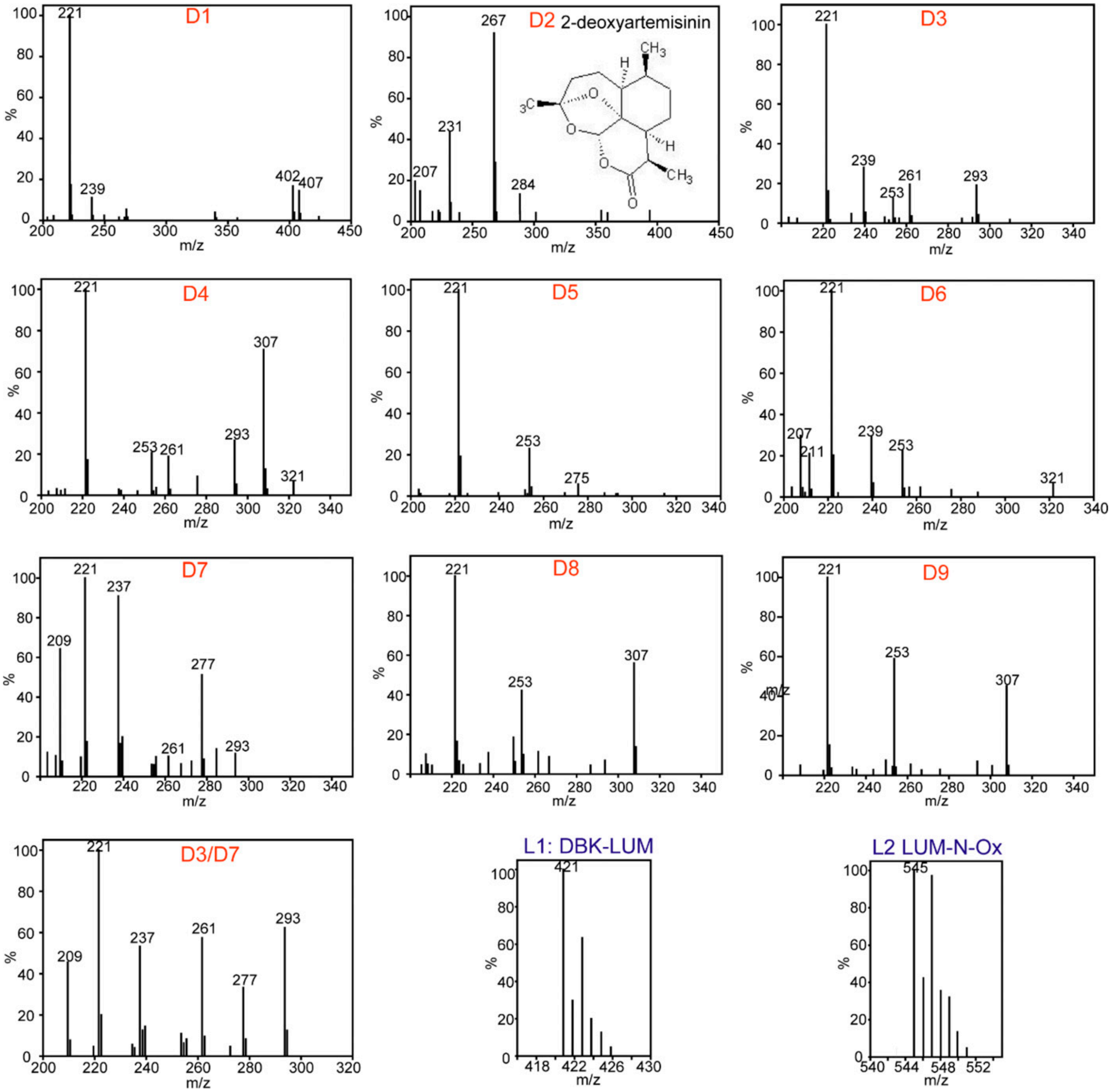

Supplemental Figure 3. AS/AQ, AM/LUM, and DHA/PIP tablets were artificially degraded at 60°C. The mass spectra for the major degradation products consistently identified are shown. D1–D2 and D3–D6 are the major AS and AM degradation products, respectively. D2, D3, and D7–D9 are degradation products of DHA. L1 and L2 are desbutylketo derivatives of LUM and LUM-N-oxide, respectively, degradation products of LUM. A mass spectrum is also shown for a mixture of D3/D7. AS/AQ = artesunate/amodiaquine; AM/LUM = artemether/lumefantrine; DHA/PIP = dihydroartemisinin/piperaquine.

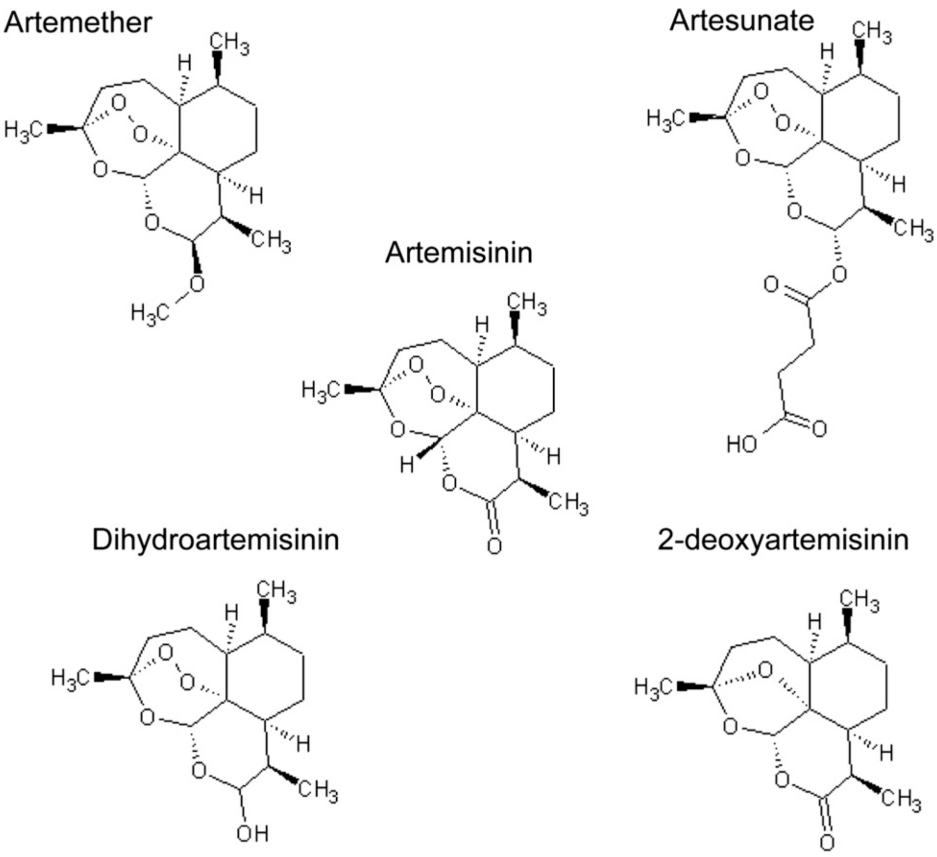

Supplemental Figure 4. Structures of artemisinin, its therapeutic derivatives (including β-artemether, α-artesunate, and dihydroartemisinin), and 2-deoxyartemisinin—a degradation product of artesunate and dihydroartemisinin.

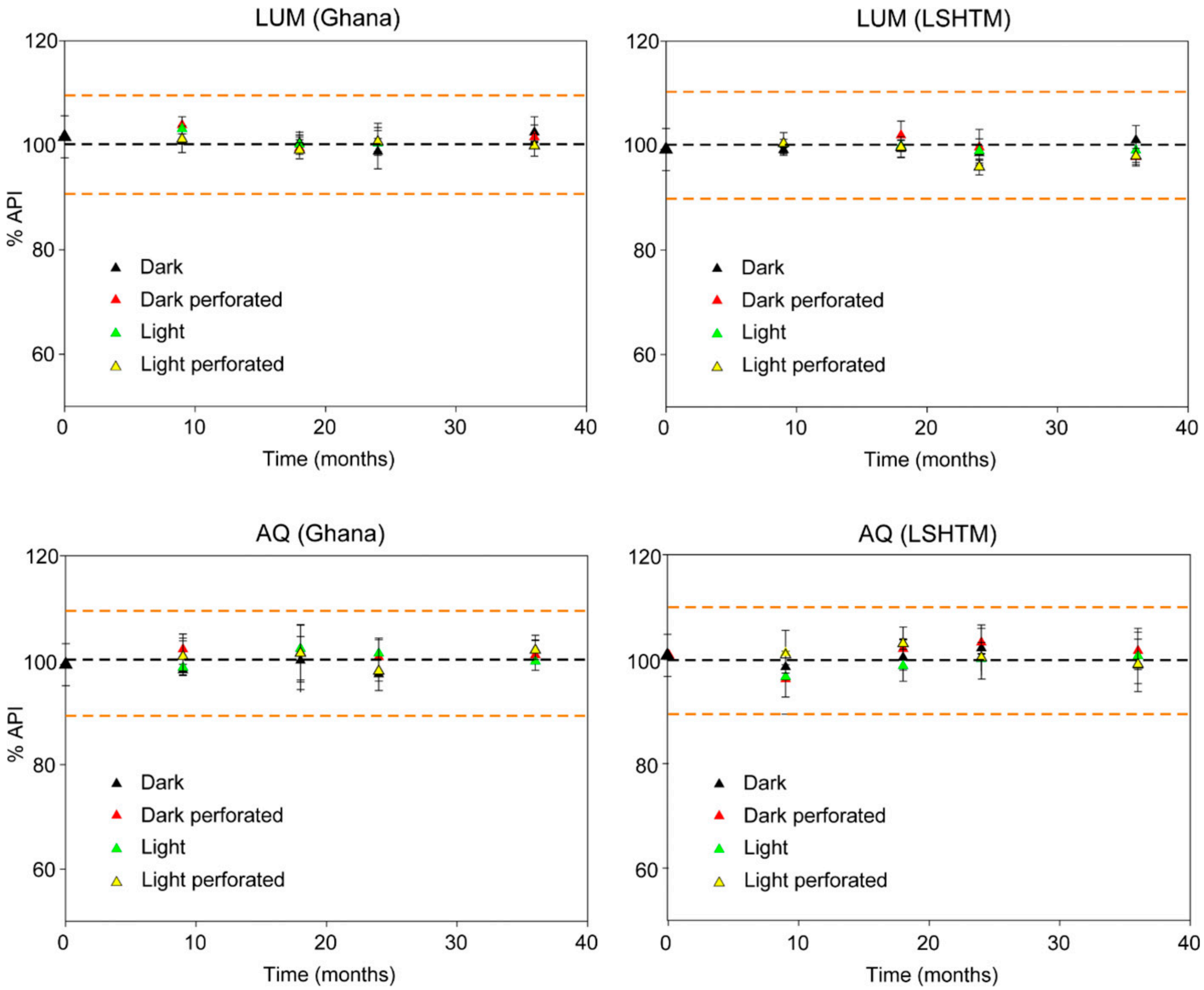

Supplemental Figure 5. Coartem® AM/LUM and Winthrop® AS/AQ samples were aged for 36 months in a stability chamber (LSHTM) with temperature and humidity settings corresponding to concurrent conditions of a field site in Kintampo, Ghana. Samples were periodically removed, and %API (LUM and AQ) was quantified using high-performance liquid chromatography photo diode array. Results are shown for tablets stored in the dark or light and with intact or perforated packaging. Error bars show standard deviation (N = 36). Orange dashed lines show acceptable range (90–110 %API) according to the International Pharmacopoeia. AM/LUM = artemether/lumefantrine; AS/AQ = artesunate/amodiaquine; API = active pharmaceutical ingredient; LSHTM = London School of Hygiene and Tropical Medicine.

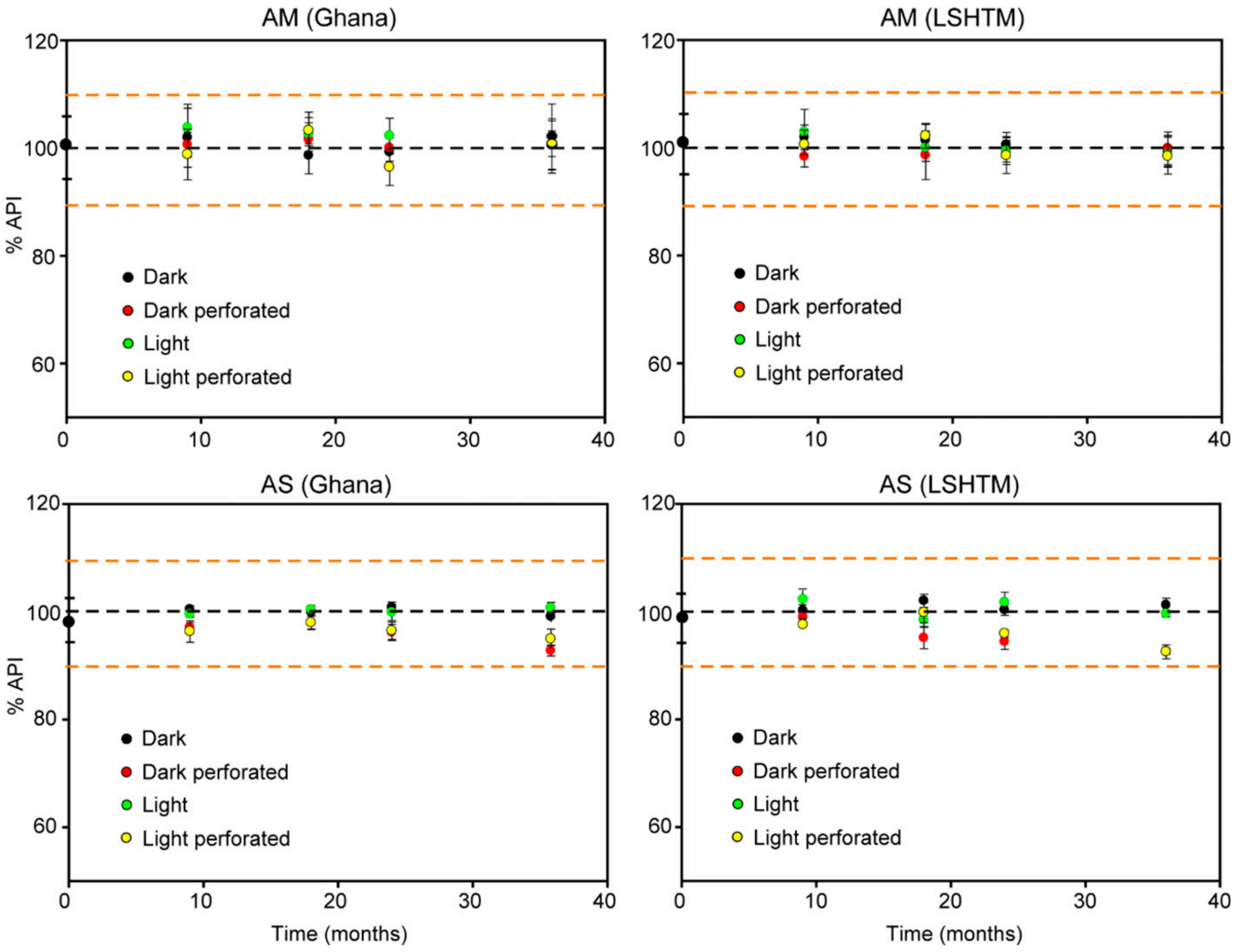

Supplemental Figure 6. Coartem® AM/LUM and Winthrop® AS/AQ samples were aged for 36 months in a stability chamber (LSHTM) with temperature and humidity settings corresponding to concurrent conditions of a field site in Kintampo, Ghana. Samples were periodically removed, and %API (AM or AS) was quantified using high-performance liquid chromatography photo diode array. Results are shown for tablets stored in the dark or light and with intact or perforated packaging. Error bars show standard deviation (N = 36). Orange dashed lines show acceptable range (90–110 %API) according to the International Pharmacopoeia. AM/LUM = artemether/lumefantrine; AS/AQ = artesunate/amodiaquine; API = active pharmaceutical ingredient; LSHTM = London School of Hygiene and Tropical Medicine.

Supplemental Table 1

IC_{50} values for antiplasmodial activity assays on test compounds using two strains of *Plasmodium falciparum*

Compound	Strain HL1204 (chloroquine sensitive)	Strain HL1210 (chloroquine resistant)
Controls		
Chloroquine	19.8 nM	138 nM
Artesunate	6.6 nM	3.4 nM
Pyrimethamine	12,200 nM	9,300 nM
Test compounds		
Artesunate	5.2 nM	5.2 nM
Artemether	3.4 nM	3.4 nM
D1*	9,036 nM	5,103 nM
D2*	2,429 nM	1,993 nM
D3*	8,869 nM	5,085 nM
D4*	2,759 nM	20,900 nM
9,10-Anhydroartemisinin	10.6 nM	7.1 nM
2-Deoxyartemisinin	Not tested	1,099 nM

IC_{50} values of ~10 nM or less are typically required for compounds to be of clinical interest.
*Used an estimated molecular weight 282 g/mol (artemisinin).

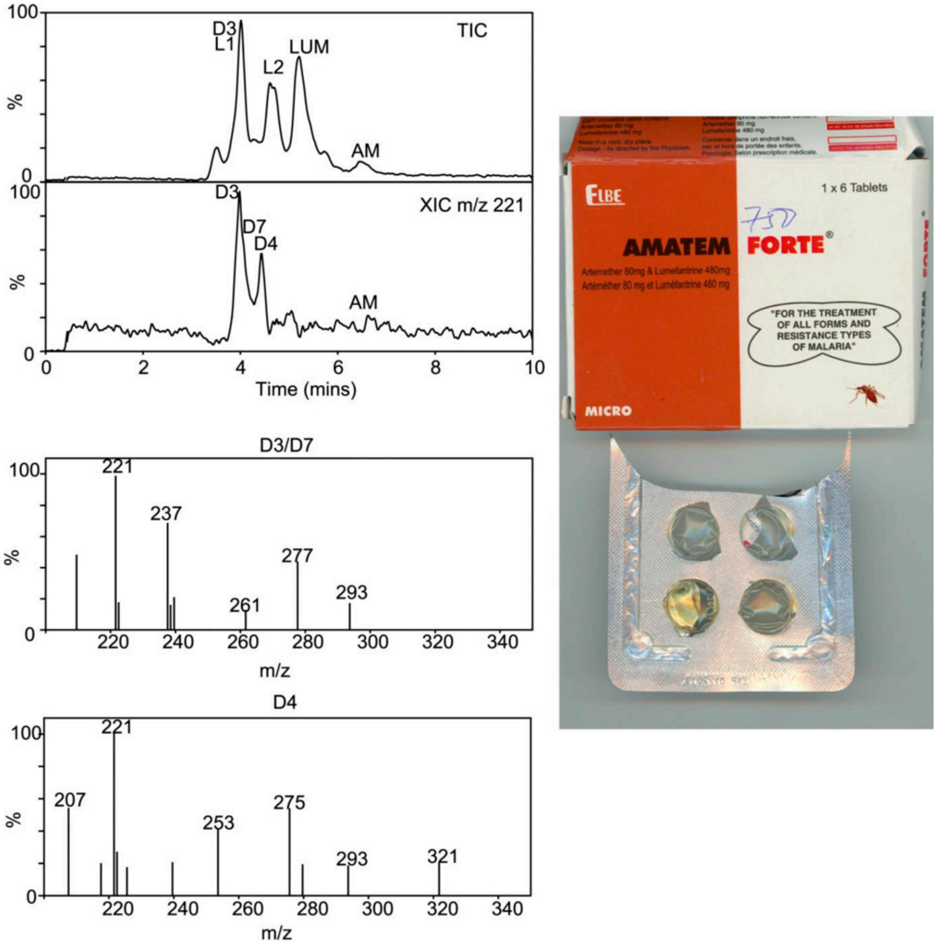

Supplemental Figure A1. Liquid chromatography mass spectrometry analysis of artemether/lumefantrine (AM/LUM) Amatem Forte® tablet reveals similar degradation products to Coartem® AM/LUM tablets artificially degraded. Extracted ion chromatograms for *m/z* 221 (signature fragment ion) are shown below the total ion chromatogram. Interestingly, a mixture of D3 and D7 (seen previously in dihydroartemisinin "forced degradation") was observed, in addition to D4. Examination of the Amatem Forte packaging revealed a sticky residue inside the blister pack.

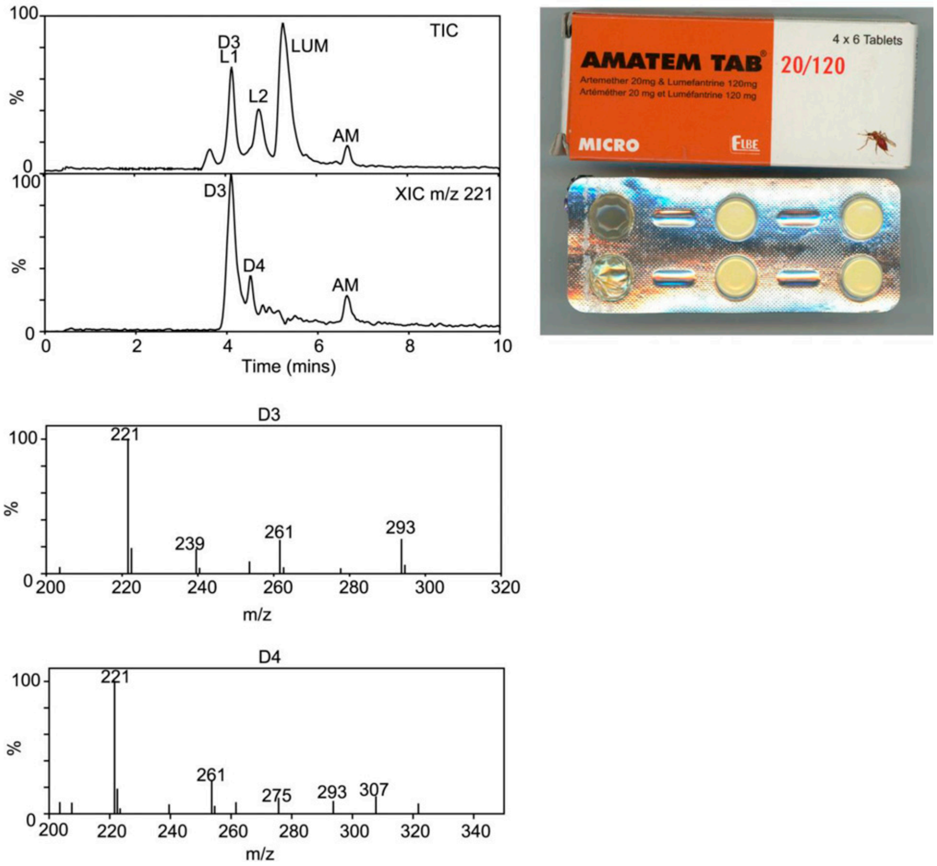

SUPPLEMENTAL FIGURE A2. Liquid chromatography mass spectrometry analysis of artemether/lumefantrine (AM/LUM) Amatem Tab® reveals similar degradation products to Coartem® AM/LUM tablets artificially degraded. Examination of the packaging revealed a sticky residue inside the blister pack.

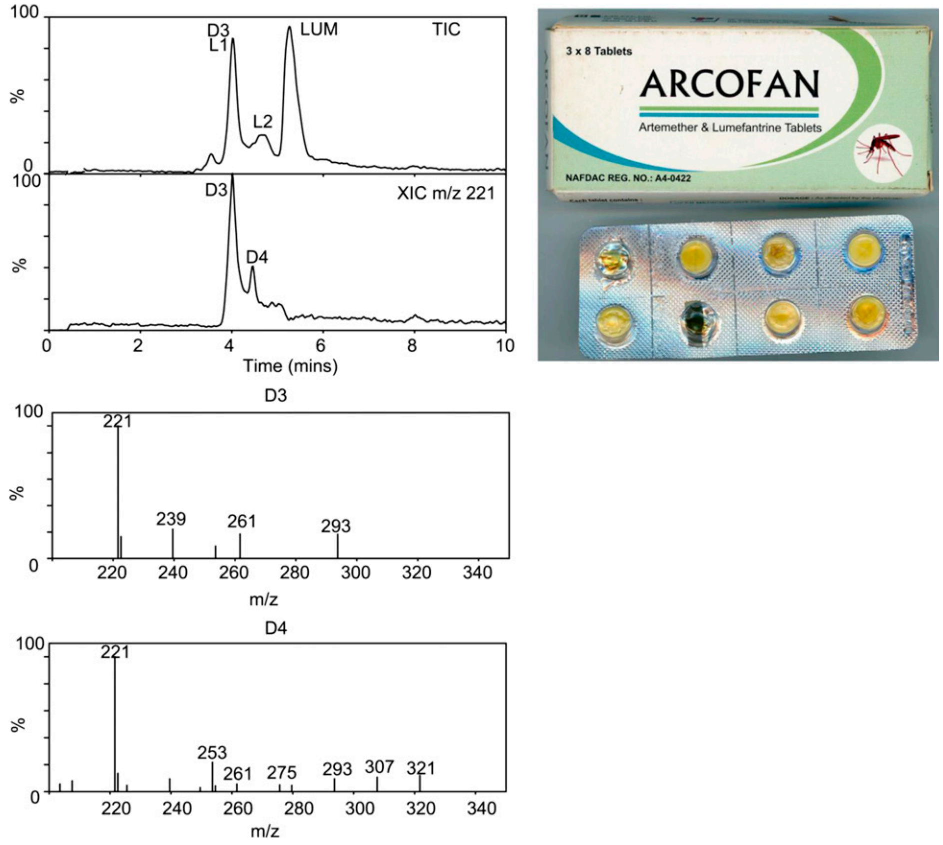

Supplemental Figure A3. Liquid chromatography mass spectrometry analysis of artemether/lumefantrine (AM/LUM) Arcofan tablet reveals similar degradation products to Coartem® AM/LUM tablets artificially degraded. Examination of the packaging revealed a sticky residue inside the blister pack, and tablets were discolored.

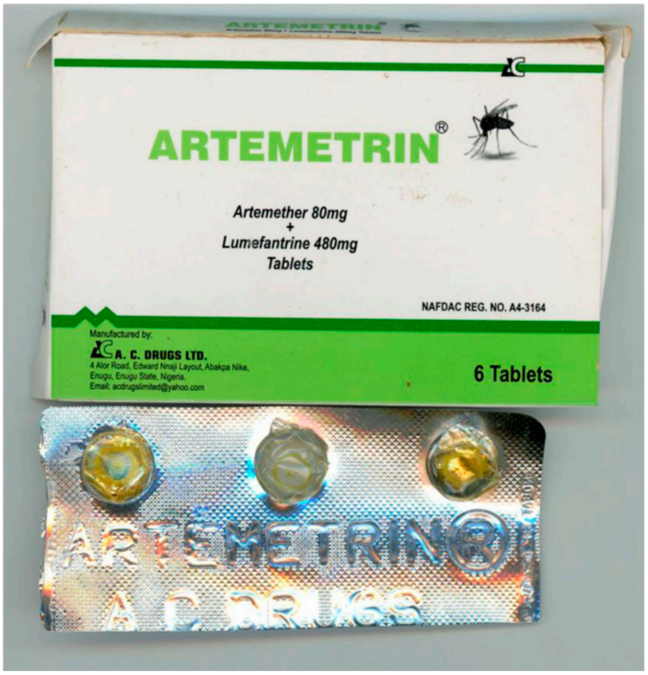

Supplemental Figure A4. No tablets remained for Artemetrin®, and therefore liquid chromatography mass spectrometry analysis was not performed. However, the packaging revealed a sticky residue inside the blister pack.

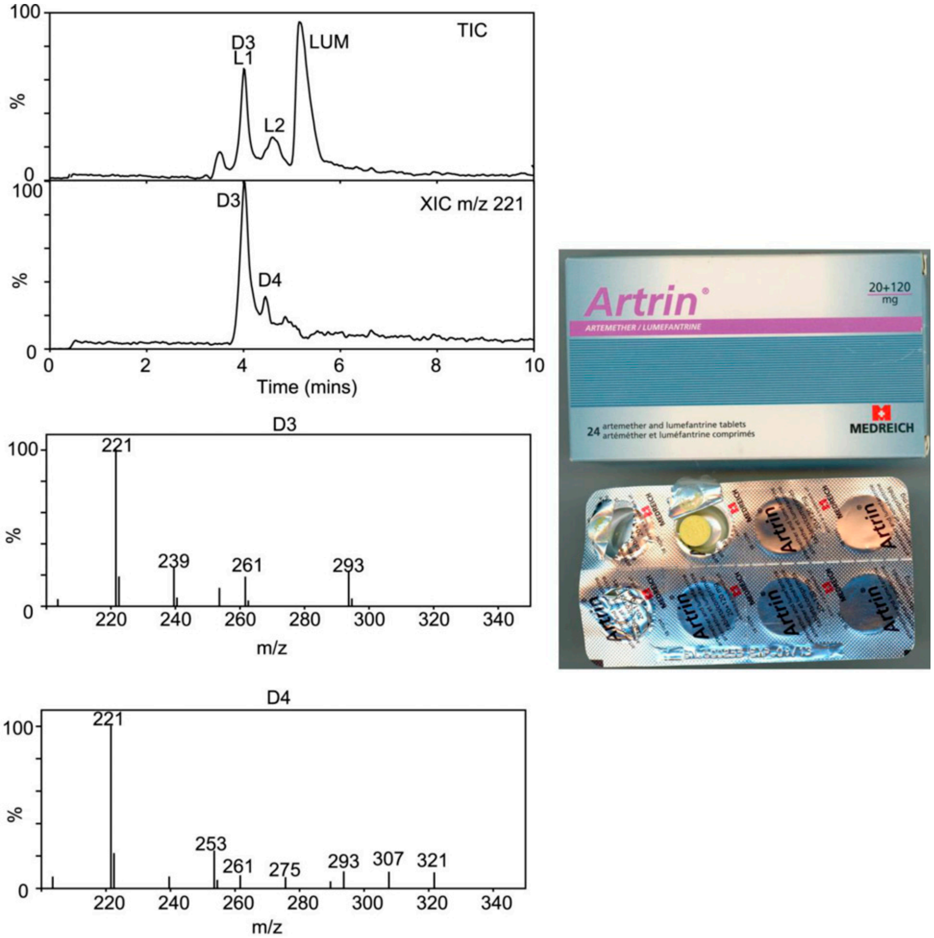

Supplemental Figure A5. Liquid chromatography mass spectrometry analysis of artemether/lumefantrine (AM/LUM) Artrin® tablet reveals similar degradation products to Coartem® AM/LUM tablets artificially degraded. Examination of the packaging revealed a sticky residue inside the blister pack.

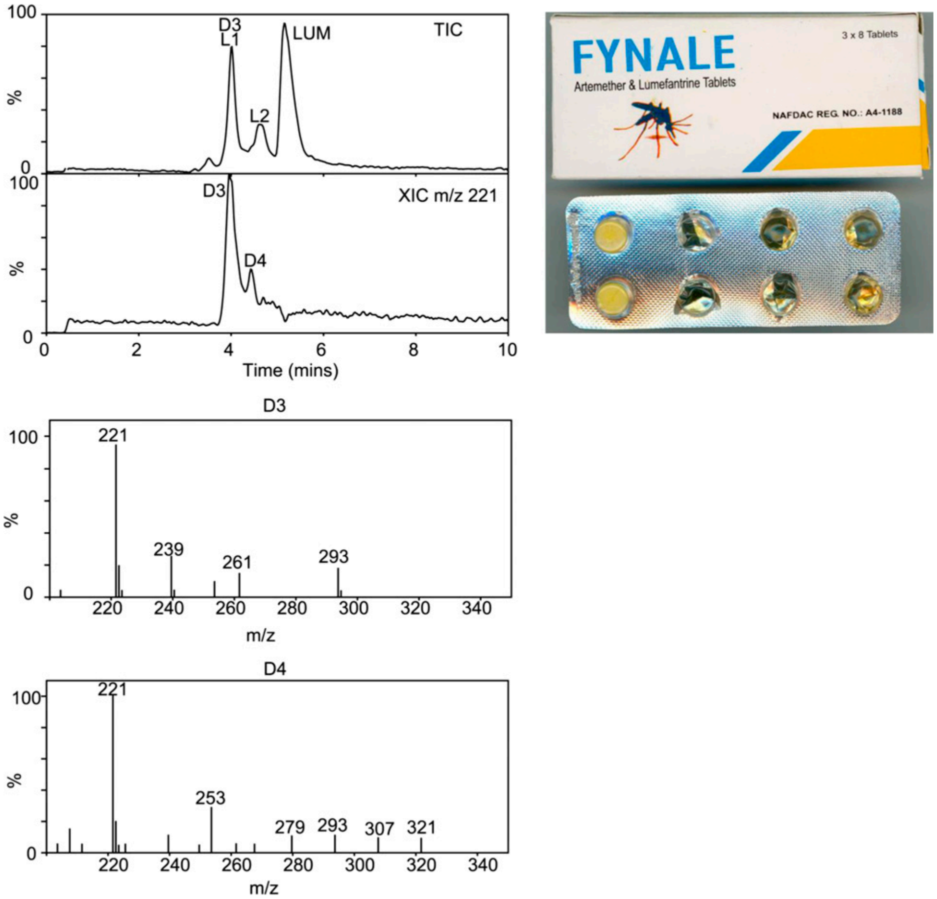

Supplemental Figure A6. Liquid chromatography mass spectrometry analysis of artemether/lumefantrine (AM/LUM) Fynale tablet reveals similar degradation products to Coartem® AM/LUM tablets artificially degraded. Examination of the packaging revealed a sticky residue inside the blister pack.

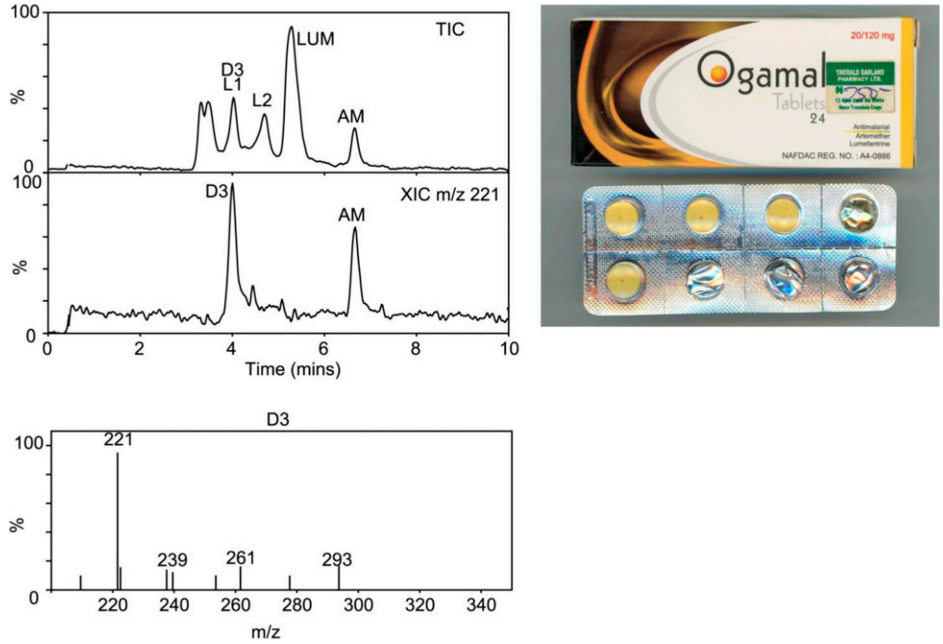

Supplemental Figure A7. Liquid chromatography mass spectrometry analysis of artemether/lumefantrine (AM/LUM) Ogamal tablet reveals similar degradation products to Coartem® AM/LUM tablets artificially degraded. Examination of the packaging revealed a sticky residue inside the blister pack, and tablets were discolored.

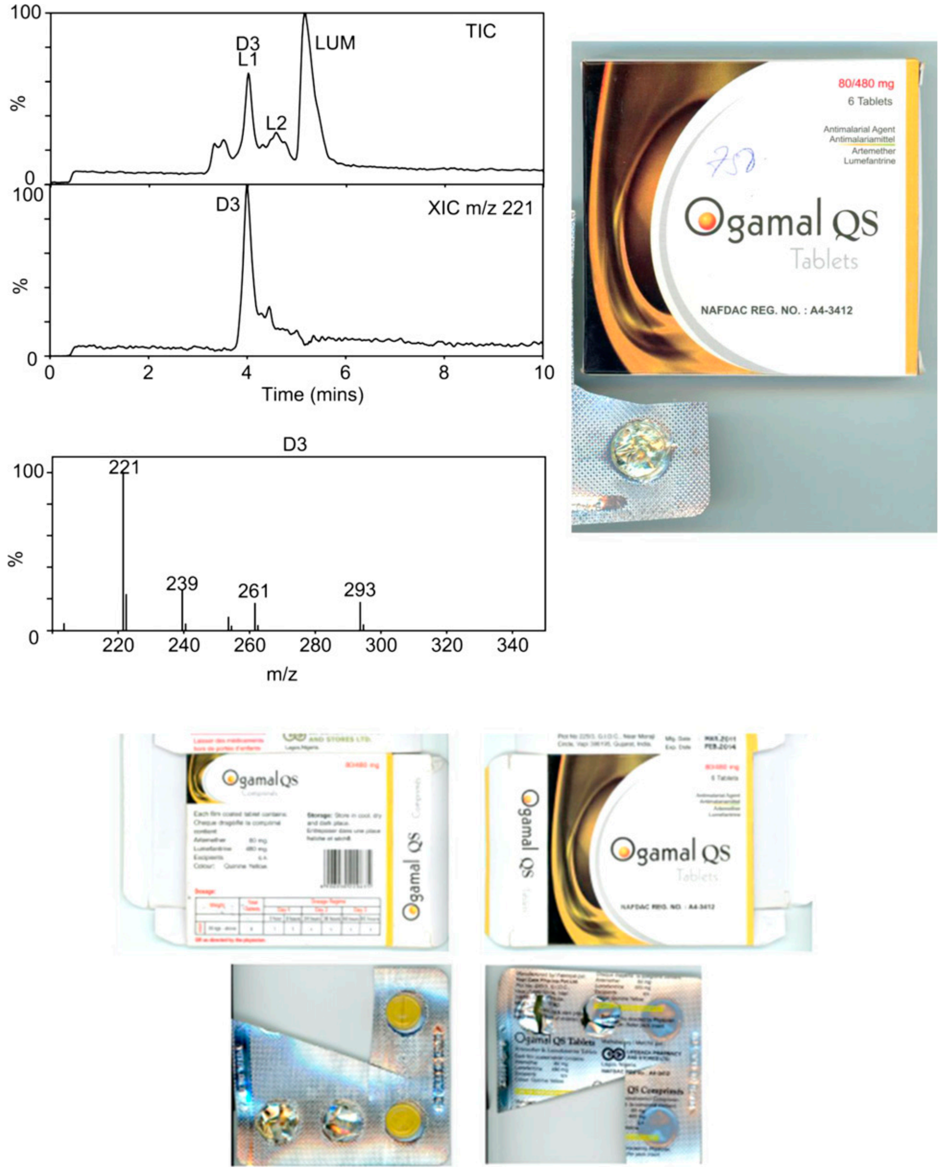

Supplemental Figure A8. Liquid chromatography mass spectrometry analysis of artemether/lumefantrine (AM/LUM) Ogamal QS tablet reveals similar degradation products to Coartem® AM/LUM tablets artificially degraded. Examination of the packaging revealed a sticky residue inside the blister pack, and tablets were discolored.

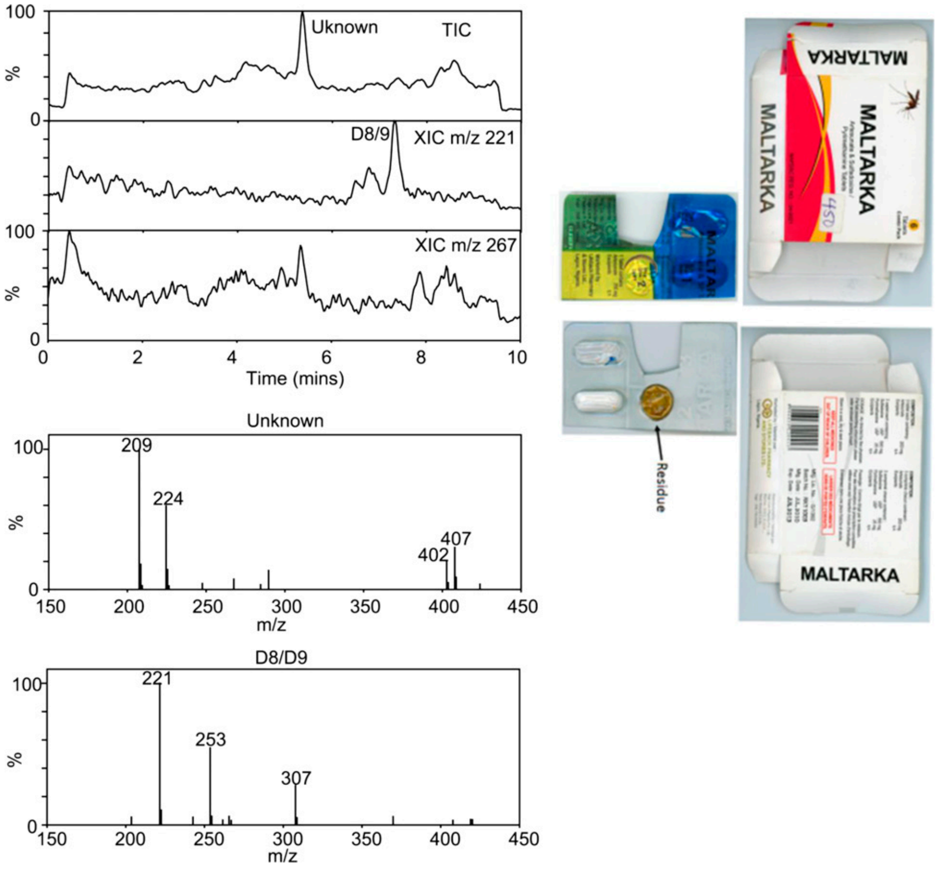

Supplemental Figure A9. Liquid chromatography mass spectrometry analysis of artesunate Maltarka tablet reveals similar degradation products to dihydroartemisinin/piperaquine tablets (Waipa) artificially degraded (D8/D9) and a previously unseen degradation product. Examination of the packaging revealed a sticky residue inside the blister pack, and tablets were highly discolored and brittle.

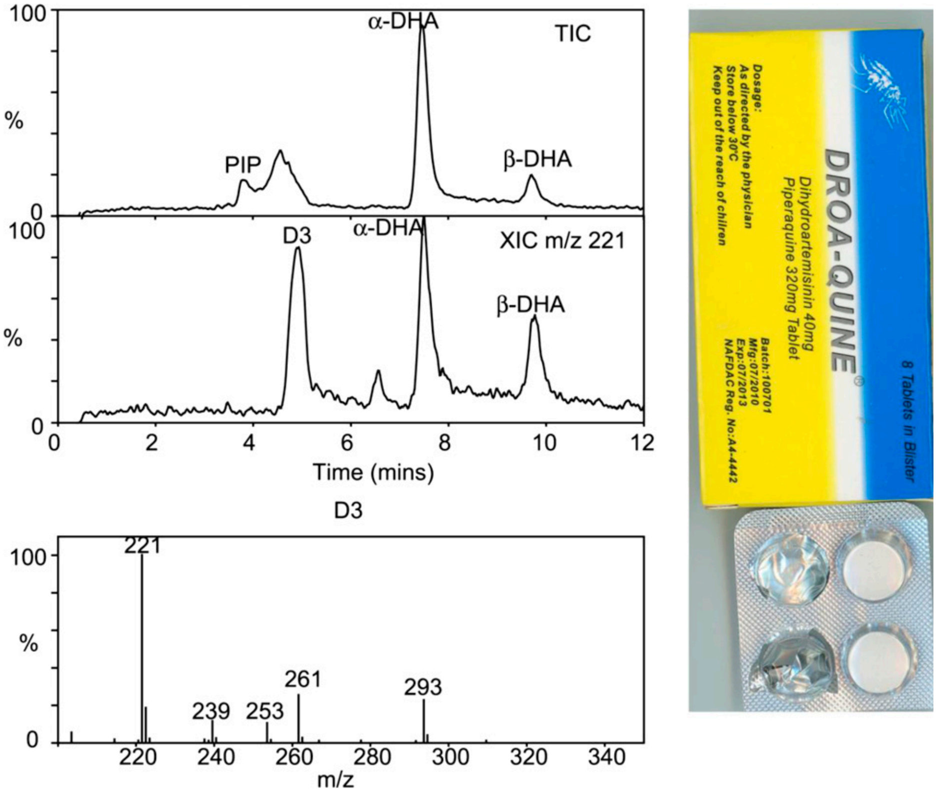

Supplemental Figure A10. Liquid chromatography mass spectrometry analysis of Droa-Quine® dihydroartemisinin/piperaquine (DHA/PIP) tablet reveals similar degradation products to DHA/PIP tablets (Waipa) artificially degraded.
